# Advancing Reversed-Phase Chromatography Analytics of Influenza Vaccines Using Machine Learning Approaches on a Diverse Range of Antigens and Formulations

**DOI:** 10.3390/vaccines13080820

**Published:** 2025-07-31

**Authors:** Barry Lorbetskie, Narges Manouchehri, Michel Girard, Simon Sauvé, Huixin Lu

**Affiliations:** 1 Center for Oncology, Radiopharmaceuticals and Research, Biologic and Radiopharmaceutical Drugs Directorate, Health Products and Food Branch, Health Canada, Ottawa, ON K1A 0K9, Canada; 2 Science Strategy and Services Innovation, Chief Technology Office Branch, Shared Services Canada, Montreal, QC H9P 1J3, Canada; narges.manouchehri@ssc-spc.gc.ca

**Keywords:** influenza, hemagglutinin, vaccine evaluation, reversed-phase, chromatography, machine learning, supervised learning, classification

## Abstract

One concern in the yearly re-formulation of influenza vaccines is the time-consuming manufacturing of vaccine potency reagents, particularly for emergency responses. The continuous evaluation of modern techniques such as reversed-phase (RP) chromatography is an asset for streamlining this process. One challenge with RP methods, however, is the need to re-optimize methods for antigens that show poor separation, which can be highly dependent on analyst experience and available data. In this study, we leveraged a large RP dataset of influenza antigens to explore machine learning (ML) approaches of classifying challenging separations for computer-assisted method re-optimization across years, products, and analysts. **Methods**: To address recurring chromatographic issues—such as poor resolution, strain co-elution, and signal absence—we applied data augmentation techniques to correct class imbalance and trained multiple supervised ML classifiers to distinguish between these peak profiles. **Results**: With data augmentation, several ML models demonstrated promising accuracy in classifying chromatographic profiles according to the provided labels. These models effectively distinguished patterns indicative of separation issues in real-world data. **Conclusions** Our findings highlight the potential of ML as a computer assisted tool in the evaluation of vaccine quality, offering a scalable and objective approach to chromatogram classification. By reducing reliance on manual interpretation, ML can expedite the optimization of analytical methods, which is particularly needed for rapid responses. Future research involving larger, inter-laboratory datasets will further elucidate the utility of ML in vaccine analysis.

## 1. Introduction

In order to mitigate the spread of influenza virus, vaccination remains the best public health strategy to prevent outbreaks. Every year, the strain composition of seasonal vaccines must be updated to match circulating viral strains [1]. As a result, the process of influenza vaccine standardization—from surveillance to reagent production to final manufacture—is a 6–8 month global endeavor [2,3]. Extensive experience has established the predictability of seasonal influenza vaccines. However, significant challenges arise when influenza viruses undergo antigenic shift, which creates novel strains with the potential to cause pandemics [4] that lead to significant disease burden and economic losses. Production bottlenecks that are manageable during seasonal vaccine manufacturing become critical when rapid pandemic responses are needed [1,3].

One such example of a bottleneck involves the production of reagents for the Single Radial Immunodiffusion (SRID) assay, which is the gold-standard for measuring immunologically active hemagglutinin (HA) and, consequently, vaccine potency. Limited reagents can take months and several groups have proposed orthogonal methods to assess potency in the event of pandemic situations [5]. Both antibody-independent [6,7,8] and antibody-dependent [9,10,11] methods have been proposed. One approach involves reversed-phase (RP) liquid chromatography (LC), which is well-established in the biopharmaceutical industry as a robust, reproducible, easily calibrated, and quantifiable physicochemical technique. With RP chromatography of multivalent influenza vaccines, the HA1 subunits of HA can be separated based on their strain without the need to develop strain-specific antibodies [12]. Since the HA1 subunit is a key target for neutralizing antibodies against influenza virus infection, the quantitation of well-folded strain-specific HA1 is valuable for vaccine quality assessments [13]. One limitation to RP separation is that assay conditions are inherently denaturing, which does not inform us on HA structural integrity. However, sample pre-treatments have been used to enrich conformationally active HA [6,14], which presents a promising approach for assessing potency. RP methods can also be rapidly adapted and optimized across formulations and vaccine modalities, unlike antibody-based methods, which offer limited flexibility when binding is disrupted [15].

For RP methodology to be widely adopted, implementing standard testing and optimization processes will be essential. In most years and products, HA1 antigens are readily separated with a single RP method [13]. However, method optimization is required for challenging cases, such as when strains co-elute or excipient formulations interfere with separation [13]. This is particularly challenging at scale for influenza vaccines, as drug product composition is complex and fixed, so samples can not be reformulated to remove interfering components. In addition, given the numerous products and formulations available, it would be impractical for a single lab to provide optimization parameters for all vaccines. Quality control laboratories, where most routine testing occurs, are also not conducive to extensive experimentation. Thus, a centralized database of analytical results and predetermined method parameters would be valuable for individual labs evaluating optimization strategies. Similar database-driven approaches have been successfully applied in other contexts [16,17].

In our laboratory, we frequently leverage a database of influenza vaccine chromatograms in this way to evaluate RP method performance across diverse samples and formulations. However, as with all cumulative databases, increasing data volume makes it increasingly difficult to identify chromatograms relevant to specific issues without proper annotation. Currently, chromatographic analyses depend heavily on manual interpretation, which significantly limits throughput. An automated strategy to classify chromatograms by separation issues would be highly valuable.

Fortunately, chromatographic data are highly structured, reproducible under consistent method parameters, and rich in information. This quantitative nature makes chromatographic data well-suited to automated analysis using advanced statistical approaches and machine learning (ML) [18,19]. In recent years, ML has emerged as a transformative tool across various scientific disciplines, including analytical chemistry. By enabling the automated analysis of complex and large-scale datasets, such as chromatograms, ML facilitates the identification of patterns and predictions at scales that would be challenging through traditionally manual methods [16]. Some previously explored applications of ML in chromatographic analysis include predicting small-molecule retention times [20,21,22], improving method development approaches [23,24], or assessing peak quality in LC mass spectrometry analysis [25,26,27]. In these applications, full chromatograms are typically not analyzed because key features like analyte retention time can be extracted. However, for evaluating issues in biotherapeutic analysis, relevant information can emerge at any point of the chromatogram.

Thus, in this study, we chose to investigate well-established dimensionality-reduction approaches and classification algorithms for full chromatogram analysis. As a fundamental supervised ML technique, classification models are trained to map input features to their corresponding labels, allowing for the accurate prediction of classes for new, unseen data. This approach is well-suited for tasks where categorizing complex data into distinct classes is informative, such as identifying chromatographic profiles associated with challenging separations.

The objective of this study was to develop a workflow for automatically classifying high-quality separations from poorly separated peaks. Although we focused on influenza analysis for its broader global impact, this workflow could be test to any routine analyses collected in quality control labs. We began this study by curating a chromatogram database containing both high-quality HA separations and poor HA separations with well-characterized sources from our laboratory. These chromatograms were collected over a decade of RP analysis on diverse samples, and their relevance to influenza vaccine analysis is presented. These challenging separations include issues such as strain compositions that co-elute, unique excipient formulations that cause HA insolubility, and novel modalities. We then labeled these data with either ‘good’ or one of the following three types of separation issues: ‘low resolution’, ‘co-elution’, or ‘no signal’. Since data for challenging separations are typically less rigorously collected than for successful ones, we also explored various sampling and augmentation methods to balance the dataset. Principal component analysis (PCA) was used to reduce the dimensionality of the data. Finally, using this database, we tested nine classification algorithms in categorizing chromatograms by separation issue. Given the increasing accessibility of chromatographic datasets and familiarity with ML approaches in cross-disciplinary applications, we believe this study will serve as a foundation for a database of shared knowledge that can ultimately improve influenza vaccine analysis.

## 2. Materials and Methods

### 2.1. Chemicals

Analytical reagent grade chemicals were used in this study. Tween 80, 1,4-dithiothreitol (DTT), trifluoroacetic acid (TFA), and Triton X-100 were obtained from Sigma-Aldrich (St. Louis, MO, USA). Acetonitrile (ACN) and 2-propanol were purchased from Merck KGaA (Darmstadt, Germany). Deionized and distilled water was produced by a Nanopure Diamond™ system (Barnstead International, Dubuque, IA, USA).

Reversed-Phase Liquid Chromatography (RPLC) analysis was performed on a Waters Alliance 2695 High-Performance Liquid Chromatography (HPLC) system coupled to a Waters 2475 Multichannel Fluorescence Detector with an 8 µL flow cell. Fluorescence of intrinsic tryptophan was collected at wavelengths $\lambda_{\mathrm{ex}}=280\;\mathrm{nm}/\lambda_{\mathrm{em}}=335\;\mathrm{nm}$. Data acquisition was performed with Empower 3 Chromatography Data Software from Waters.

### 2.2. Sample Preparation

Various samples obtained from commercial vendors were used. These contained influenza antigen in both quadrivalent and trivalent formulations, typically in split or subunit formats at 30 µg/mL HA of each strain. Samples were prepared in polypropylene vials to minimize adsorption. For a typical preparation [13,28], 5 µL of 400 mM DTT (final 20 mM) and 5 µL of 1.0% *w*/*v* Tween 80 (final 0.05% *w*/*v*) were added to an aliquot of 90 µL influenza antigen sample, which was then heated in boiling water for three minutes. For samples treated with trypsin, 1 µL of TPCK-treated trypsin (1 mg/mL) was added to 50 µL of a sample and incubated at 37 °C for 25–30 min. The sample was then treated with 10 µL of 10% zwittergent, 5 µL of 400 mM DTT, 5 µL of 1% Tween, and 29 µL of PBS. The resulting 100 µL sample was boiled for three minutes.

### 2.3. Conditioning and Separation

Several instrumentation methods were used in this study, and they are described in the Supplementary Information. Typically, mobile phase A contained water with TFA ranging from 0.04% to 0.1%, and mobile phase B contained 25% ACN and 75% 2-propanol, with TFA ranging from 0.03% to 0.1%. Reductions in TFA concentration provided minor improvements to the separation of HA1 peaks. Flow rates were always set to 1 mL/min, and autosampler temperature was set at 5 °C. A list of columns and gradients used in this manuscript is shown in Supplementary Table S1.

For analyses with the MICRA^®^ HPLC NPS-ODSI, 33 mm × 4.6 mm, 1.5 µm particles column (Eprogen, Darien, IL, USA), chromatographic separations were carried out with the gradient parameters shown in Supplementary Tables S2 and S3. Mobile phase A contained 0.04% (*v*/*v*) aqueous TFA, and mobile phase B contained 0.03% (*v*/*v*) TFA in 25% ACN and 75% 2-propanol.

For analyses with the PLRP-S 300 Å, 8 µm, 250 mm × 4.6 mm column (Agilent Technologies, Santa Clara, CA, USA), chromatographic separations were carried out with the gradient parameters shown in Supplementary Table S4. Mobile phase A contained 0.1% (*v*/*v*) aqueous TFA, and mobile phase B contained 0.1% (*v*/*v*) TFA in 25% ACN and 75% 2-propanol.

### 2.4. Data Processing

Chromatograms were exported from Empower as text files (.arw extensions) with associated instrument, method, and sample metadata. These were further processed with Python. Chromatograms were filtered by fluorescence detection and associated with metadata such as method name, sample name, and manufacturing year. Manufacturing year was associated with the strain composition. Unrelated injections were removed by sample name (for example, ‘zero injections’). For 6 products, the final dataset contained 672 chromatograms over 3 instrument methods (Methods A, B, and C in Supplementary Table S1) and 8 seasons. Separation issues were manually labeled with 3 groups of known issues—co-elution, no signal, low resolution—or labeled with ‘good’ for acceptable separation. Fluorescence intensity was then transposed into a dataframe with metadata. Most chromatograms were collected with a sampling rate of 10 pts/s. Higher or lower sampling rates removed. Data and associated analyses were stored on the Canada Federal Science Data Hub (https://www.canada.ca/en/shared-services/services/tools-science.html, accessed on 28 July 2025).

### 2.5. Machine Learning

To develop a robust classification model for chromatograms of influenza antigens, we implemented a ML pipeline including data preprocessing, dimensionality reduction, and classifier evaluation. The primary objective was to accurately categorize chromatograms into four distinct classes: ‘good’, ‘low resolution’, ‘co-elution’, and ‘no signal’.

Given the class imbalance in the dataset, we tried to tackle this challenge with over- and under-sampling [29]. However, these methods did not yield satisfactory results, as they led to models with inflated accuracy that failed to generalize well to unseen data. To address this, we employed data augmentation techniques [30] to synthetically enhance the dataset, particularly focusing on the minority classes. The augmentation methods included Gaussian noise addition, intensity scaling, time shifting, and peak spiking. These augmentation strategies aimed to enrich the training data, enabling the models to learn a more diverse set of patterns and improve classification performance across all classes.

PCA was then applied to the augmented dataset to reduce the number of features and computational complexity [31].

The preprocessed data was used to train and evaluate nine different supervised ML classifiers [32], including Random Forest, Support Vector Machine, Logistic Regression, K-Nearest Neighbors, Decision Tree, Naive Bayes, Gradient Boosting, XGBoost, and AdaBoost. Each model was trained using a 70–30 train–test split, and was evaluated using 5-fold cross-validation to ensure robustness. Performance metrics, including accuracy, precision, recall, and F1 score, were computed to assess and compare the effectiveness of each classifier in accurately categorizing the chromatograms.

## 3. Results

### 3.1. Data Collection and Key Features

**Figure 1:**
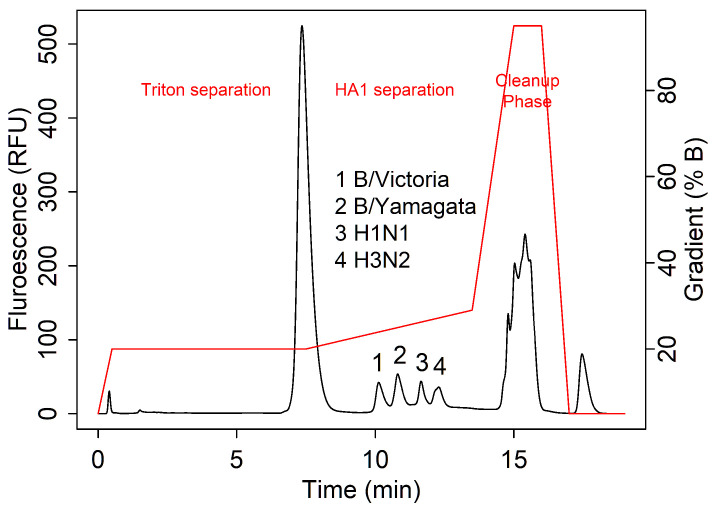
A typical chromatogram showing HA1 antigen separation in a quadrivalent influenza sample. The gradient is displayed in red, and the elution order of all HA1 strains are labeled.

From 2005 to 2023, data were collected on a diverse variety of influenza antigens by RP methods. These samples ranged from monovalent to quadrivalent, incorporating a variety of vaccine-relevant excipients such as Triton X-100, β-propiolactone, thiomersal, and α-tocopherol. Both fluorescence and absorbance data were collected, but only fluorescence data were analyzed due to its increased sensitivity [28]. Samples were reduced by DTT, denatured by boiling, and solubilized using Tween-80 prior to separation on an Eprogen MICRA^®^ HPLC NPS-ODSI column (33 mm × 4.6 mm, 1.5 µm particles). Table 1 shows an overview of this database and the key method characteristics that were tested. Figure 1 shows an example of the parts in a standard RPLC chromatogram for influenza analysis.

Out of the 22 samples tested, some were re-formulated annually with WHO-recommended strains and were re-analyzed to establish whether changes affected method performance. When we encountered challenging separations in new samples, these historical data provided the basis for further optimization. Injections with similar sample characteristics and peak profiles could then be used to infer potential underlying issues. The next three sections give examples of three known issues seen in our laboratory, which were used as the basis of a dataset for classification.

### 3.2. Examples of Challenging Separations

#### 3.2.1. Excipient Interference

In drug products, excipients are often included as non-active ingredients to enhance stability and efficacy. Since these compounds do not directly contribute to potency, bioanalytical RP methods are typically designed to separate excipient peaks from those of the active drug. These interferences can be identified manually and optimized when excipient peaks are clearly visible, such as those found with Triton X-100 [13]. However, challenges arise when interactions between excipient and active drug prevent analysis. Excipients can affect the higher-order structures of proteins [33], leading to aggregation or denaturation that disrupts separation.

**Figure 2:**
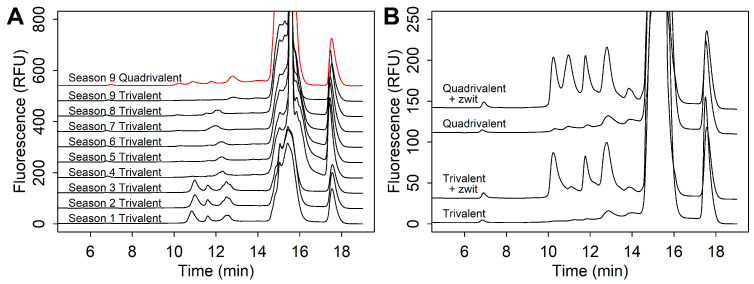
(**A**) Chromatograms of a trivalent and quadrivalent samples that show poor resolution, with standard sample preparation following a season where a formulation shift was recorded. (**B**) Chromatograms showing good antigen separation of both quadrivalent and trivalent samples after treatment with zwittergent detergent.

One example of excipient impact was observed in a trivalent sample over years of analysis. We saw separation between HA peaks significantly diminished starting from one specific season (Figure 2A). At first, the underlying issue in resolution quality was not clear. However, over time, a persistent change in the clean-up phase of chromatogram (from 13.5–16 min) also suggested a concomitant change in excipients (Figure 2A). A review of excipients showed the addition of ingredients, such as α-tocopherol and ethanol, after this season, which were also present in another unrelated quadrivalent sample that contained similar patterns in separation and peak shapes (Figure 2A). These results were consistently observed over multiple seasons and lots, while other sample formulations were unaffected.

In later seasons, we found that poor HA separation in these samples could be rescued with the addition of zwittergent (Figure 2B). In the SRID assay, zwittergent detergent was often used for HA solubilization by the dissociation of aggregates. With zwittergent added at 1% *w*/*v*, three peaks were clearly seen in the trivalent sample and four peaks in the quadrivalent sample.

#### 3.2.2. Strain Co-Elution

**Figure 3:**
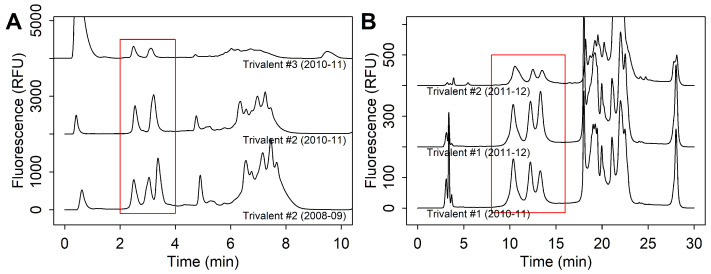
(**A**) Chromatograms showing strain co-elution when trivalent samples were analyzed in the 2010–2011 season with the Eprogen MICRA^®^ HPLC NPS-ODSI column (33 mm × 4.6 mm, 1.5 µm particles) column compared to the 2008–2009 when strain co-elution was not observed. (**B**) Chromatograms showing that analysis with the PLRP column allowed for HA1 separation in both the 2010–2011 and 2011–2012 seasons. Red boxes indicate HA1 peaks.

One issue that is common to all products during strain re-formulations is the potential of peak co-elution between the H1N1, H3N2, B/Victoria, and B/Yamagata strains. This was first observed in the 2010–2011 season, where trivalent products were recommended to contain an A/California/7/2009 (H1N1)-like virus, an A/Perth/16/2009 (H3N2)-like virus, and a B/Brisbane/60/2008-like virus. In this season, trivalent samples were observed with two peaks by RPLC instead of the three peaks seen in previous years (Figure 3). This issue was observed in all trivalent samples of that season, regardless of excipient formulation and lot. When monovalent samples were analyzed individually, peaks corresponding to HA1 from the H1N1 strain and the H3N2 had the same retention time. This issue was also observed in the 2011–2012 season, which used the same WHO strain recommendations for the Northern Hemisphere. In subsequent years, when recommendations changed, peak co-elution was not observed.

To resolve this issue, other columns were tested with trivalent samples from the 2010–2011 and 2011–2012 season to improve resolution of HA1 peaks. The PLRP column was able to separate strains in samples from both seasons (Figure 3B). Interestingly, the PLRP column did not require an alternative method for Triton X-100 separation, which was needed in the NPS-ODSI column. Instead, Triton X-100 was eluted in the clean-up phase, and showed no interference with HA1 peaks.

#### 3.2.3. Recombinant Modalities

**Figure 4:**
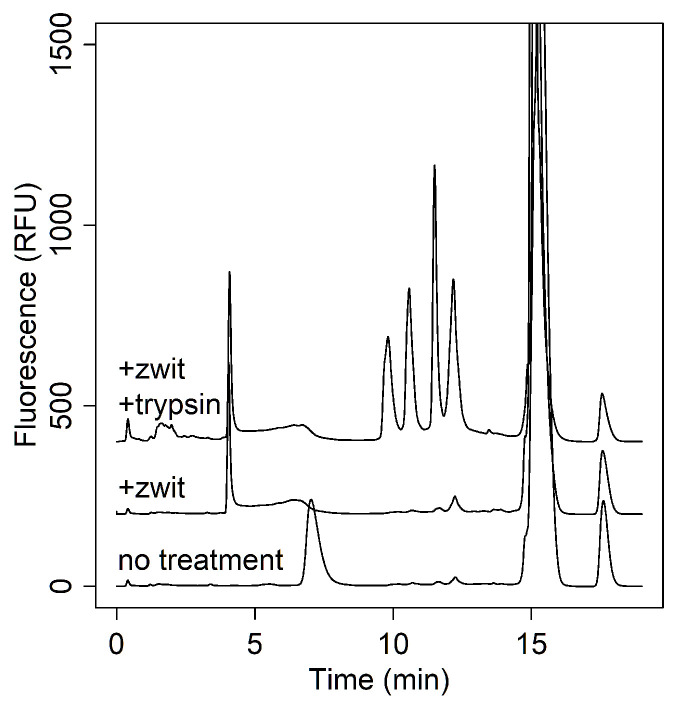
Chromatograms showing RPLC separation of a quadrivalent recombinant sample with no treatment (bottom), zwittergent detergent (middle), and zwittergent with trypsin digest (top).

An increasing challenge for influenza analysis is the appearance of novel vaccine modalities [34]. Recombinant products were one such promising innovation to accelerate the production process. These products typically present HA as a single polypeptide chain where HA1 and HA2 domains cannot be separated by disulfide reduction. This can be problematic for RPLC analysis, as the highly hydrophobic transmembrane domains of HA2 prevent the clean separation of full-length HA peaks (Figure 4, bottom trace). Even with zwittergent treatment, full-length HA could not be eluted (Figure 4, middle trace).

Previously, Wen et al. used trypsin to selectively digest HA content that was altered by stress conditions, leaving only native pre-fusion HA1 available for analysis by RPLC [6]. In an effort to cleave HA1 from HA2, we also applied trypsin to the full-length HA polypeptide sequence. This approach enabled the clean separation of HA1 strains, consistent with the peak profile observed in RP analysis of inactivated influenza vaccines (Figure 4, top trace).

### 3.3. Machine Learning for Chromatographic Classification in Vaccine Quality Assessment

Investigation into the root causes of these challenging separations often requires extensive manual interpretation of multiple influenza seasons and products in order to establish elution patterns. However, as data accumulates, this manual interpretation becomes increasingly time-consuming and limited in scalability. When rapid responses are needed, reducing method optimization times becomes crucial. To address this, we explored the potential of using ML as a robust, automated solution for efficiently processing and classifying large volumes of chromatographic data.

To demonstrate this proof of concept, we filtered the original dataset of 13,454 by fluorescence detection, three instrument methods, and sample name. Unrelated injections, such as zero injections, were removed. Manufacturing year was manually annotated, and can be used to infer strain composition. The resulting filtered dataset contained 627 chromatograms analyzing six samples over 9 years. Since analyses over multiple instrument methods and injection volumes were included, the dataset reflects the variability that an analyst would realistically encounter in practice. The fully annotated dataset can be found in the Supplementary Data.

Upon manual review, each chromatogram was labeled according to four visually distinguishable patterns which reflect the underlying quality of antigen separation, as identified in previous sections: ‘good’, ‘low resolution’, ‘co-elution’, and ‘no signal’. Ideal or ‘good’ chromatograms will feature clearly separated HA1 peaks, either three or four depending on the sample valency. In contrast, chromatograms with ‘low resolution’ separation will appear as indistinct peaks with overlapping signals. Chromatograms labeled with ‘co-elution’ show distinct but fewer-than-expected peaks, with two peaks as a clear indicator of strain co-elution. Finally, extremely low or undetectable HA peaks suggest hydrophobic antigens that are not well-retained by the column method.

**Figure 5:**
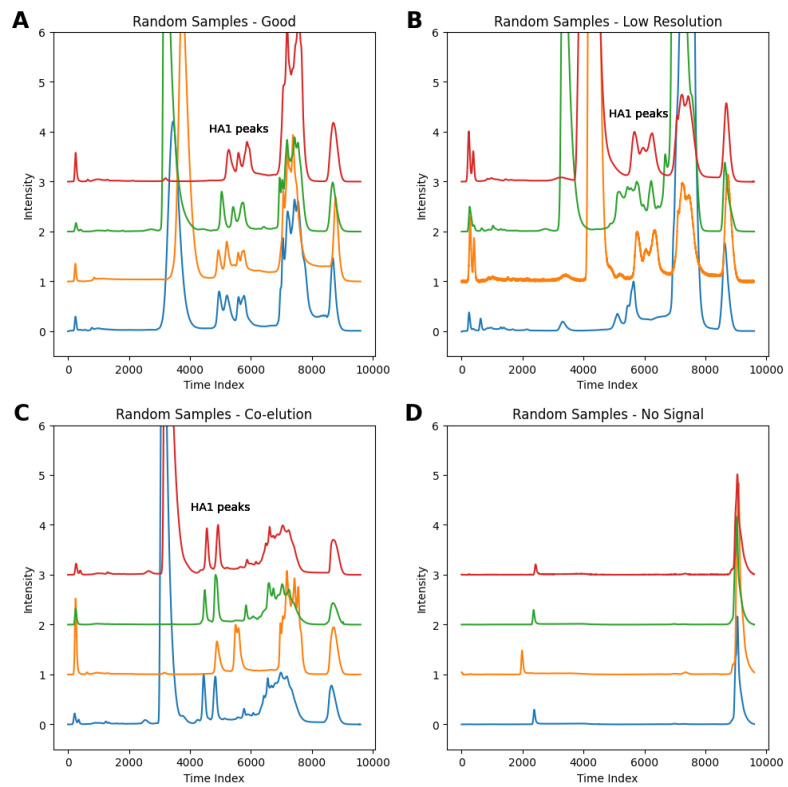
Chromatograms within the dataset showing (**A**) good separation, (**B**) low-resolution separation, (**C**) co-elution of peaks, and (**D**) a low or undetectable HA1 signal.

Figure 5, shows a range of each chromatogram type within the filtered dataset containing 627 chromatograms.

**Figure 6:**
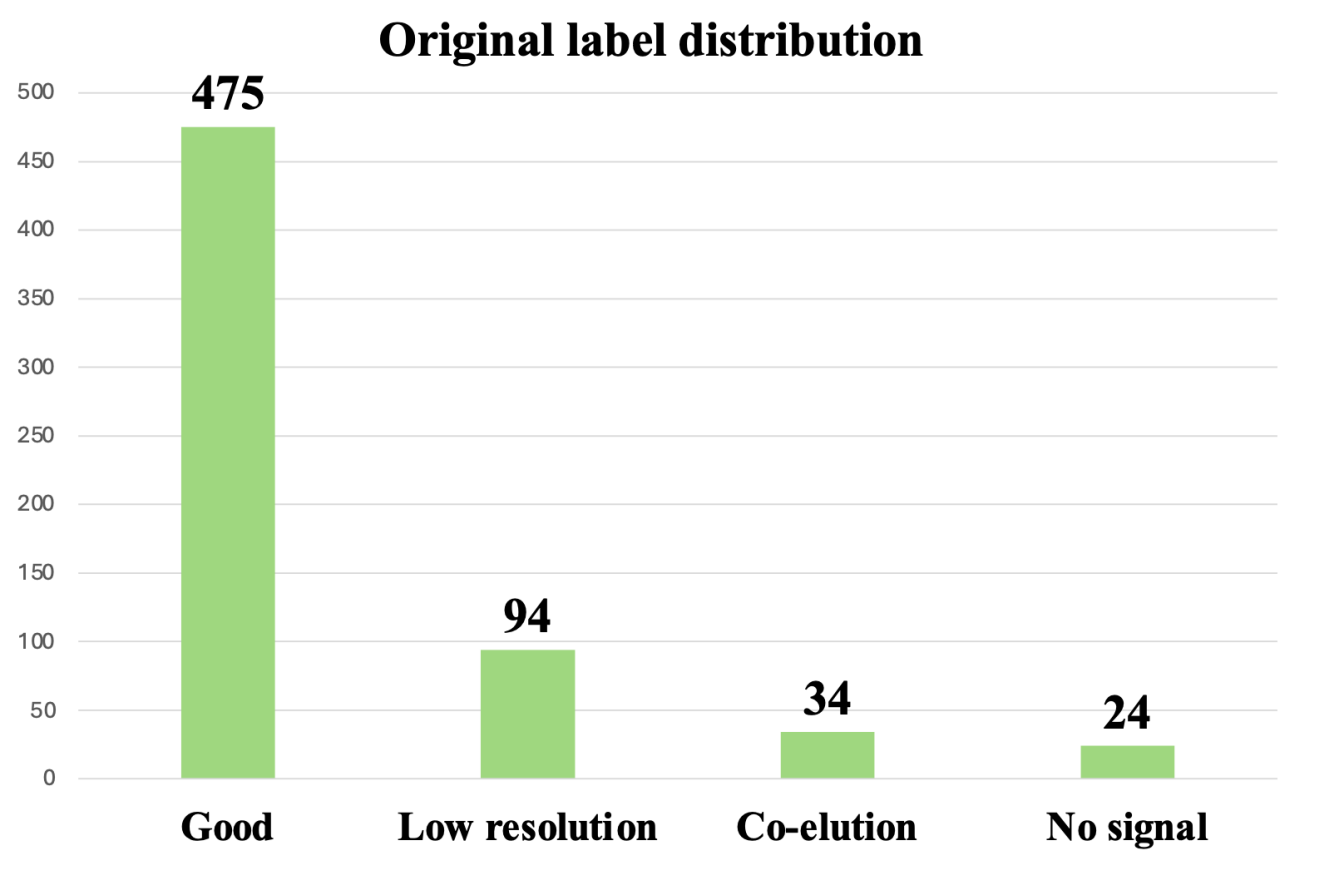
Distribution of labels in the filtered dataset, composed of 627 chromatograms.

As shown in Figure 6, the ‘good’, ‘low resolution’, ‘co-elution’, and ‘no signal’ chromatograms contained 475, 94, 34, and 24 instances, respectively, highlighting the severe under-representation of the latter three groups. In quality-control environments, this kind of imbalance is common, since analyses that fall outside of specifications are often not repeatedly evaluated.

#### 3.3.1. Oversampling and Undersampling in Addressing Class Imbalance

In our initial approach to mitigate the class imbalance present in our chromatogram dataset, we employed resampling techniques, specifically oversampling and undersampling [29]. Oversampling involves duplicating instances of minority classes to balance the dataset, while undersampling reduces the number of instances in the majority class.

Despite achieving high accuracy metrics during training and validation phases, we observed that these resampling techniques did not yield reliable performance on real-world, imbalanced data. The models tended to favor the majority class, and the high accuracy was misleading, as it did not reflect the model’s ability to correctly classify minority-class instances.

Recognizing these shortcomings, we explored alternative methods to address the class imbalance without compromising the integrity of the dataset.

#### 3.3.2. Data Augmentation for Addressing Class Imbalance

To tackle the challenge of data imbalance, we implemented a data augmentation strategy that involved generating synthetic variations of the existing chromatograms [30]. Four transformations were applied to the filtered dataset containing 627 chromatograms in order to simulate experimental variability. These four strategic preprocessing techniques and data augmentation methods include the following:

- Gaussian noise: Adds random noise sampled from a Gaussian distribution to simulate signal variability and improve model generalization.
- Intensity scaling: Randomly scales the intensity of the chromatogram to mimic variations in signal strength and enhance model robustness.
- Time shifting: Shifts the chromatogram along the time axis by a random amount to account for temporal variations in retention times.
- Peak spiking: Distorts the intensity of a randomly selected point in the chromatogram to simulate signal irregularities.

**Figure 7:**
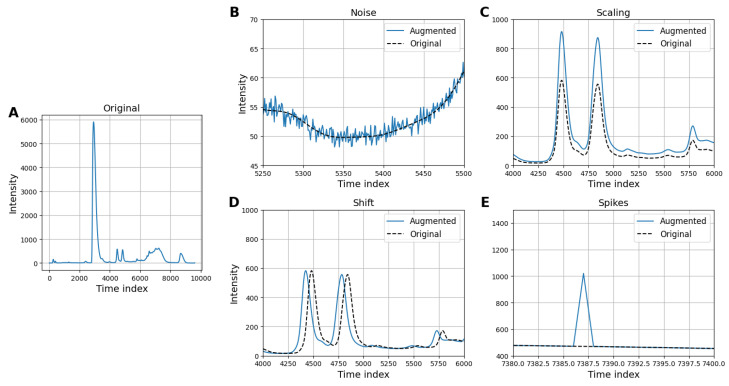
Examples of a chromatogram showing co-elution and its augmented variants. (**A**) The original chromatogram is compared to chromatograms augmented with (**B**) Gaussian noise, (**C**) intensity scaling, (**D**) time shifting, and (**E**) peak spiking.

This approach aimed to enrich the diversity of the minority classes with with variability that was experimentally relevant, which would provide the model with a more representative set of examples to learn from. By augmenting the data in this manner, we aimed to improve the model’s capacity for generalization in order to achieve a more balanced and reliable classification performance across all classes. In Figure 7, we illustrate samples from the underrepresented classes and the augmented versions using the four methods.

**Figure 8:**
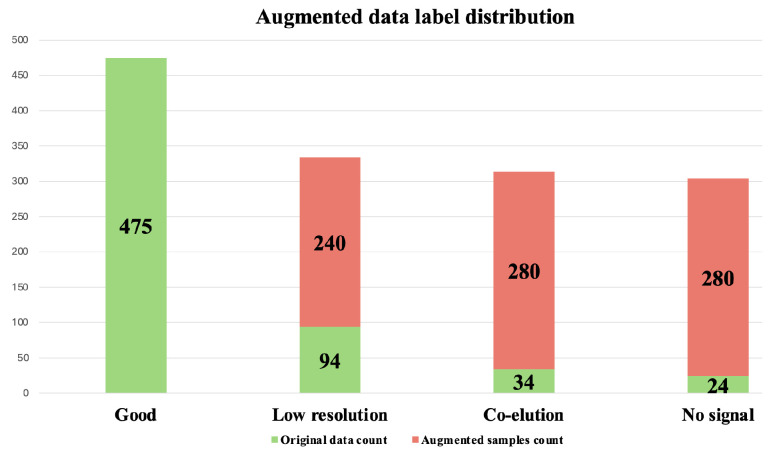
Distribution of labels in the augmented dataset, which contained a total of 1472 chromatograms. Green represents chromatograms that appear in the filtered dataset of 627 chromatograms. Red represents chromatograms generated through augmentation of the filtered dataset, totaling 845 chromatograms.

After applying these four transformations to the filtered dataset containing 627 chromatograms, an additional 845 chromatograms were generated. Combining the filtered dataset with the augmented chromatograms resulted in a dataset containing 1472 chromatograms. In Figure 8, we show the distribution of observations after data augmentation. This augmented dataset containing 1472 chromatograms was used for further analysis.

#### 3.3.3. PCA for Dimensionality Reduction

**Figure 9:**
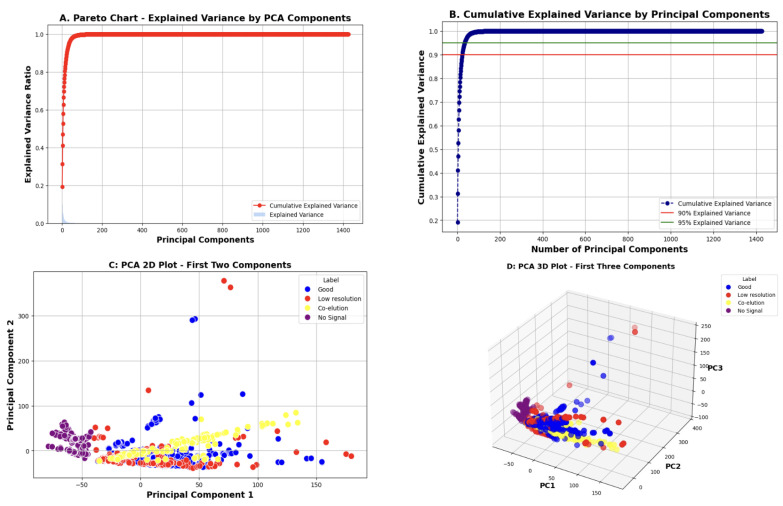
PCA results. (**A**) Pareto chart showing the variance explained by each individual principal component. (**B**) Cumulative explained variance plot indicating how much variance is retained as more components are included. (**C**) A 2D PCA scatter plot using the first two components, showing class separation. (**D**) A 3D PCA scatter plot using the first three components for a more comprehensive view of class distributions in reduced dimensions.

To manage the high dimensionality of the augmented dataset and reduce computational complexity, we applied PCA [31] across entire chromatograms to retain the most informative features. PCA is a common approach for dimensionality reduction in chromatograms [19,35]. In Figure 9, we illustrate the following plots to evaluate PCA results.

- The Pareto chart displays the amount of variance explained by each principal component, which helps determine which components are most informative.
- The cumulative explained variance plot shows how variance accumulates as more components are added. This helps select the optimal number of components to retain while balancing accuracy and complexity.
- The 2D and 3D PCA plots project the data onto the first respective two and three principal components to visualize class separability.

Based on the cumulative explained variance, we determined that retaining 34 components preserves 95% of the total variance, capturing a meaningful amount of information from the original data.

#### 3.3.4. Classification, Model Performance, and Results

In this study, our objective was to identify chromatograms based on peak profiles without requiring manual interpretation. To achieve this, we applied supervised classification to an augmented dataset reduced by PCA. In this dataset, each full chromatogram was paired with a label indicating its class, which enables the models to learn relationships between input features and class labels. This would enable the models to accurately predict the classes of new, unseen chromatograms without manual peak picking or expert interpretation.

To train and evaluate our models effectively, we divided the dataset into training and testing subsets using a 70–30 split, where 70 percent of the augmented data (n = 1472) was used for training and 30 percent for testing. Table 2 summarizes the performance of each classifier.

Classification performance on testing datasets, with augmented data improved significantly over oversampling and undersampling techniques. Several models, such as Random Forest, Gradient Boost, and XGBoost, achieved >95% accuracy, recall, and F1 score, demonstrating the potential of using ML algorithms in differentiating the four types of chromatograms. To further assess classification performance, we also present confusion matrices for each model in Supplementary Figure S1. These matrices provide a detailed comparison between the true and predicted classifications, highlighting each model’s strengths and limitations in distinguishing between the chromatogram classes.

These findings are promising, suggesting that data augmentation and ML can be applied to capture complex patterns in imbalanced chromatographic data, reducing the reliance on manual intervention.

## 4. Discussion

In recent years, the increasing frequency and scale of pandemic threats have renewed interest in developing more efficient influenza vaccine production processes. When the SRID assay was developed in the 1970s, its compatibility with seasonal influenza vaccines were central to its adoption [36]. At the time, long reagent manufacturing times were manageable because seasonality was predictable. However, in the wake of vaccine delays during the 2009 H1N1 pandemic [36], interest in establishing other valid assessment schemes has been renewed [5,15]. Developing an accessible methodology that can be rapidly redeveloped to suit all vaccines and potential strain formulations is a priority.

In this study, we show how RPLC methods can be rapidly adapted to a diversity of antigens with simple protocol changes, such as sample pretreatments and columns. We also show that full chromatograms are amenable to automated ML classification, provided that the data are carefully balanced and that dimensionality is reduced. Data augmentation led to significantly better performance compared to oversampling or undersampling methods.

This proof-of-concept workflow addresses the challenge of manual analysis amid constantly growing data in chromatographic databases. These could be curated databases centralized for shared use amongst other laboratories [16,17] or simply chromatograms from routine QC analysis collected during a drug product’s life cycle. In this study, we focused on challenges specific to influenza vaccine analysis because any assay developed for potency would require global coordination. However, the labels used in this study can be extended to other issues of interest in QC labs. For example, the detection of system contaminants, ghost peaks, and drifting baselines. Analysts can be trained to identify any of these issues; however, manual interpretation can be highly subjective, especially when real-world data does not match their training sets. Having a systematic and objective method for chromatogram evaluation would be valuable.

This work demonstrates that RP methodology for influenza vaccine analysis is highly adaptable to modern data analysis approaches, unlike SRID. Future efforts will focus on evaluating unsupervised ML algorithms to minimize manual labeling, as well as incorporating larger, inter-laboratory datasets to assess scalability.

The next challenge to adopting RPLC for influenza potency analysis is the assessment of stability-indicating properties correlated to immunogenicity [6,14]. This potential, coupled with the amenability of RP to rapid optimization and automated analysis, could position RPLC as a valuable component in the development of robust and generalizable platforms for fast influenza vaccine potency assessment.

## 5. Conclusions

Since the 1980s, the SRID assay has proven its utility over decades of experience globally [34]. Developing a reproducible assay for a complex multi-antigenic sample, such as the influenza vaccine, is challenging. As technology matures, however, novel approaches will become increasingly advantageous for improving vaccine access and efficiency.

With RPLC, methods can be rapidly adjusted and data could be programmatically analyzed at-scale. With the promise of coupling RPLC to potentially stability-indicating sample pre-treatments, we hope that this study will provide the foundation for establishing a scalable approach for influenza vaccine potency analysis—an essential need for emergency scenarios.

## Figures and Tables

**Table 1:** Summary of influenza analysis parameters. *: not including zero and blank injections.

Variable	Value
Years	2009–2023
No. of products	22
Reference standard sources	NIBSC, TGA, CBER
No. injections *	13,454
No. influenza A strains analyzed	18
No. influenza B strains analyzed	8
Influenza A subtypes	H1N1; H3N2; H7N9;
Influenza B lineages	Victoria; Yamagata;
Columns used	15
Methods	438
Mobile phase A	Water, 0.04–0.1% TFA
Mobile phase B	25% ACN, 75% 2-propanol, 0.03% to 0.1% TFA
Injection volumes	0.5–150 µL
Column temps	25–65 °C

**Table 2:** Performance metrics of different machine learning methods.

Method	Accuracy	Precision	Recall	F1 Score
Random Forest	0.968	0.961	0.960	0.960
Support Vector Machine	0.893	0.902	0.893	0.891
Logistic Regression	0.911	0.912	0.911	0.911
K-Nearest Neighbors	0.920	0.923	0.920	0.920
Decision Tree	0.921	0.922	0.921	0.921
Naive Bayes	0.798	0.812	0.798	0.790
Gradient Boosting	0.953	0.954	0.953	0.953
XGBoost	0.960	0.960	0.960	0.960
AdaBoost	0.876	0.881	0.876	0.877

## Data Availability

Federal Open Science Repository of Canada.

## Supplementary Materials

The following supporting information can be downloaded at: https://www.mdpi.com/article/10.3390/vaccines13080820/s1, Figure S1: Confusion Matrices; Table S1: Columns and gradients used in this manuscript; Table S2: Gradient conditions for methods to separate Triton from HA peaks with the NPS-ODSI column; Table S3: Gradient conditions for standard methods to separate HA peaks with the NPS-ODSI column; Table S4: Gradient conditions for methods to separate co-eluting HA peaks with the PLRP column.

## Abbreviations

The following abbreviations are used in this manuscript:

ACN	Acetonitrile
DTT	Dithiothreitol
HA	Hemagglutinin
HPLC	High-Performance Liquid Chromatography
ML	Machine Learning
PCA	Principal Component Analysis
RPLC	Reversed-Phase Liquid Chromatography
RP	Reversed-Phase
SRID	Single Radial Immunodiffusion
TFA	trifluoroacetic acid
TPCK	L-1-tosylamido-2-phenylethyl chloromethyl ketone
