# Supplementary material for: Advancing Reversed-Phase Chromatography Analytics of Influenza Vaccines Using Machine Learning Approaches on a Diverse Range of Antigens and Formulations
Source: Vaccines (Basel). 2025 Jul 31;13(8):820. doi: 10.3390/vaccines13080820 (PMC12390064; doi:10.3390/vaccines13080820)
Supplement: Supplementary file 1 [file vaccines-13-00820-s001.zip › vaccines-3746609-supplementary.pdf]

Table S1: Columns and gradients used in this manuscript

**Method Name**	**Column**	**Temp°C**	**Purpose**
Method A	NPS-ODSI 33 mm × 4.6 mm, 1.5 µm	55	Triton separation
Method B	NPS-ODSI 33 mm × 4.6 mm, 1.5 µm	60	Triton separation
Method C	NPS-ODSI 33 mm × 4.6 mm, 1.5 µm	55	Triton separation
Method D	NPS-ODSI 33 mm × 4.6 mm, 1.5 µm	60	Standard method
Method E	NPS-ODSI 33 mm × 4.6 mm, 1.5 µm	60	Standard method
Method F	PLRP-S 300 Å, 8 µm, 250 mm × 4.6 mm	45	Resolving co-elution

Table S2: Gradient conditions for methods to separate Triton from HA peaks with the NPS-ODSI column

**Method A**		**Method B**		**Method C**	
Minutes	%B	Minutes	%B	Minutes	%B
0	5	0	5	0	5
0.5	21	0.5	20	0.5	20
5.5	21	5.5	20	7.5	20
9.5	36	10.5	30	13.5	29
12	95	12	95	15	95
13	95	13	95	16	95
14	5	14	5	17	5
16	5	16	5	19	5

Table S3: Gradient conditions for standard methods to separate HA peaks with the NPS-ODSI column

**Method D**		**Method E**	
Minutes	%B	Minutes	%B
0	20	0	18
5	36	5	36
7	95	7	95
9	95	9	95
10	20	10	18
12	20	12	18

Table S4: Gradient conditions for methods to separate co-eluting HA peaks with the PLRP column

**Method F**	
Minutes	%B
0	20
5	36
7	95
9	95
10	20
12	20

Support Vector Machine

True Label \ Predicted Label	Good	Low resolution	Co-elution	No-signal
Good	141	2	0	0
Low resolution	28	70	3	0
Co-elution	7	0	87	0
No-signal	0	0	0	91

Random forest

True Label \ Predicted Label	Good	Low resolution	Co-elution	No-signal
Good	136	7	0	0
Low resolution	5	94	2	0
Co-elution	0	0	94	0
No-signal	0	0	0	91

Logistic Regression

True Label \ Predicted Label	Good	Low resolution	Co-elution	No-signal
Good	132	10	1	0
Low resolution	15	82	4	0
Co-elution	0	3	91	0
No-signal	0	0	0	91

K-Nearest Neighbors

True Label \ Predicted Label	Good	Low resolution	Co-elution	No-signal
Good	130	13	0	0
Low resolution	7	91	2	1
Co-elution	1	1	92	0
No-signal	0	0	0	91

Decision Tree

True Label \ Predicted Label	Good	Low resolution	Co-elution	No--signal
Good	131	11	1	0
Low resolution	5	95	1	0
Co-elution	1	1	92	0
No-signal	0	0	0	91

Naïve Bayes

True Label \ Predicted Label	Good	Low resolution	Co-elution	No-signal
Good	131	5	7	0
Low resolution	23	55	23	0
Co-elution	0	1	93	0
No-signal	3	1	0	87

Gradient Boosting

True Label \ Predicted Label	Good	Low resolution	Co-elution	No-signal
Good	133	10	0	0
Low resolution	7	93	1	0
Co-elution	1	0	93	0
No-signal	0	0	0	91

XGBoost

True Label \ Predicted Label	Good	Low resolution	Co-elution	No-signal
Good	132	10	1	0
Low resolution	5	92	3	0
Co-elution	0	0	94	0
No-signal	0	0	0	91

AdaBoost

True Label \ Predicted Label	Good	Low resolution	Co-elution	No-signal
Good	126	17	0	0
Low resolution	18	78	5	0
Co-elution	2	1	91	0
No-signal	0	0	0	91

Figure S1: Confusion Matrices
